# Global Mental Health: From Science to Action

**DOI:** 10.3109/10673229.2012.649108

**Published:** 2012-02-15

**Authors:** Vikram Patel

**Affiliations:** From the Centre for Global Mental Health, London School of Hygiene & Tropical Medicine, London (United Kingdom) and Sangath, Goa (India)

**Keywords:** developing countries, global mental health, scaling up

## Abstract

This article charts the historical development of the discipline of global mental health, whose goal is to improve access to mental health care and reduce inequalities in mental health outcomes between and within nations. The article begins with an overview of the contribution of four scientific foundations toward the discipline's core agenda: to scale up services for people with mental disorders and to promote their human rights. Next, the article highlights four recent, key events that are indicative of the actions shaping the discipline: the Mental Health Gap Action Programme to synthesize evidence on what treatments are effective for a range of mental disorders; the evidence on task shifting to nonspecialist health workers to deliver these treatments; the Movement for Global Mental Health's efforts to build a common platform for professionals and civil society to advocate for their shared goal; and the Grand Challenges in Global Mental Health, which has identified the research priorities that, within the next decade, can lead to substantial improvements in the lives of people living with mental disorders. The article ends by examining the major challenges for the field, and the opportunities for addressing them in the future. (harv rev psychiatry 2012;20:6–12.)

## INTRODUCTION: THE SCIENCE

The emergence of the discipline of global mental health can be traced to a long and distinguished history of interdisciplinary research going back at least as far as Kraepelin's 1904 investigation in Java of the cross-cultural epidemiology of severe mental disorders.^1^ The original goal of this research effort was to determine the relevance of a biomedical, psychiatric perspective on mental disorders—one largely derived from observations in the cultures of Europe and North America—to other cultures and societies around the world. This effort became more intense in the 1970s, following the arrival of the phenomenologically based psychiatric classifications, which emphasized psychopathology, rather than psychoanalytical causal interpretations, as the primary basis for taxonomy and clinical decision making. Two strands of research examined the application of these classifications, one taking a dominantly relativist view of mental disorders and the other assuming a universalist perspective. The former was a derivative of a strong research endeavor to document indigenous systems of knowledge that have described mental disorders from time immemorial. Over time, it came to be clear that each approach had strengths and weaknesses, and that integrating their methodological contributions was essential for the development of a “new cross-cultural psychiatry” or a culturally sensitive psychiatry.^2^,^3^ The consensus that emerged from this integrative approach was that despite the important contextual influences on how mental disorders were experienced, explained, and acted upon, these health conditions affected people in all cultures and societies, and were neither a figment of the “Western” imagination nor a colonial export.

Having established that mental disorders were “real” causes of human suffering, four major contributions played critically important roles in consolidating the evidence that forms the foundation of the discipline of global mental health.^4^ First was the evidence demonstrating the strong associations between mental disorders and social disadvantage, especially poverty, violence, gender disadvantage, and conflicts and disasters. This evidence was first synthesized in a 1995 report, *World Mental Health*.^5^ The evidence base was updated in 2009 for the Commission for Social Determinants in Health^6^ of the World Health Organization (WHO) and in 2010 by a systematic review on poverty and common mental disorders.^7^ Second was the development of the DALY (disability-adjusted life year), a metric that reflects the contribution of a disorder or disease to disability and mortality, and the large and growing body of cross-national epidemiological research that revealed the staggering impact of mental disorders. These contributions demonstrated that mental disorders—notably, conditions such as depression, alcohol use, and schizophrenia—were leading causes of the global burden of disease and that this burden resulted from their high prevalence, chronic or relapsing course, onset early in life, and impact on disability and, to a lesser extent, mortality.^8^ A related body of evidence demonstrated the intimate interrelationships between physical health problems and mental disorders, each fueling the other and producing worse outcomes for both, leading to the slogan “no health without mental health.”^9^ Third was the body of evidence, much of it published in the past decade, demonstrating the efficacy and cost-effectiveness of a range of pharmacological and psychosocial treatments for mental disorders in low- and middle-income countries (LAMICs).^10^ Fourth and last was the evidence demonstrating the systematic denial and abuse of the basic right to a life with dignity for people with mental disorders worldwide—in particular, those living with psychoses or mental disabilities, whether residing in hospitals or living in their own communities.^11^,^12^ This prompted Kleinman, an icon of global mental health, to declare this last situation as a “failure of humanity.”^13^

Despite this robust evidence base, the reality for most people affected by mental disorders has been an uncaring health system that does little to respond to their needs, leading to estimates that up to three of four affected persons in LAMICs do not receive the treatments known to work.^14^ In sub-Saharan Africa this “treatment gap” can exceed 90%, even for schizophrenia and other psychoses, the most severe and disabling of mental disorders.^15^ This gap is hardly surprising, given that LAMICs command less than 20% of global mental health resources.^16^ This evidence was the basis for the *Lancet* series on global mental health, a series of six articles published in September 2007, which sought to focus the global health spotlight on mental disorders. The *Lancet*, the leading journal for global health, had been championing neglected and priority global health issues for several years through various series of articles. These series, typically organized by global experts in the field, have provided space for synthesizing evidence from diverse sources and an opportunity to use this evidence to call for appropriate, responsive action. The *Lancet's*, series on global mental health was pivotal in the development of the discipline.

That series was led by three editors (two academics from London-based institutions, including the author of this article, and one from the WHO), with robust support from the *Lancet*'s, editor-in-chief and an interdisciplinary, global academic advisory board. The series focused squarely on the needs and resources for addressing mental disorders (not “mental health” in its broadest sense) in LAMICs. Five articles documented the burden and impact of mental disorders, the evidence on the effective treatments for mental disorders, the unmet needs for evidence-based care in LAMICs, the grave shortages and iniquitous distribution of global mental health resources, and the barriers to scaling up services for mental disorders.^9^,^10^,^16^–^19^ Based on this evidence, the series' last article was a call to action to scale up the coverage of services for people with mental disorders.^20^ The authors recommended that this scaling-up process needed to be informed by two principles: scientific evidence on cost-effective treatments and a respect for the human rights of people affected by mental disorders. The authors also called for greater investments in building the research evidence to guide this scaling-up process.

I will now briefly review four recent events that have galvanized the discipline of global mental health and that illustrate the variety of actions needed for the discipline to flourish and, one hopes, to achieve its ambitious goals: synthesizing the global evidence on what treatments should be scaled up through primary care; building the evidence base on how these treatments should be delivered in resource-constrained settings; establishing a social movement to mobilize resources and political will; and setting the priority agenda for research. In the final section, I will consider three key barriers that the field will need to address in the years ahead and strategies on how that might be done.

## THE ACTIONS

### Synthesizing Evidence on What Should Be Scaled Up: mhGAP

The Mental Health Gap Action Programme (mhGAP) is the WHO's flagship program on mental health. It was launched in 2008 with the mandate of producing evidence-based guidelines for managing mental, neurological, and substance use disorders by nonspecialist health workers in routine health care settings. Eight groups of “priority conditions” were identified using an established set of criteria, including burden and impact. These conditions were depression, schizophrenia, and other psychotic disorders (including bipolar disorder); suicide prevention; epilepsy; dementia; disorders due to use of alcohol and illicit drugs; and mental disorders in children. A two-year process—led by a core team in WHO, supported by an international Guidelines Development Group—produced guidelines using the Grading of Recommendations Assessment, Development and Evaluation (GRADE) methodology.^21^ The guidelines were packaged together as a 100-page manual;^22^ the *mhGAP–Intervention Guide* (mhGAP-IG) was launched in October 2010 by the WHO director-general. These guidelines are a uniquely valuable resource in global mental health, as they serve to address the central question faced by global health practitioners: what can be done in routine health care, by nonspecialist health workers, to treat mental disorders. Moreover, in addressing a range of health conditions that have shared primary origins in the brain, the guidelines also break down the traditional biomedical walls separating neurology, psychiatry, and addiction. These divisions of health care, which emanate from top-down, expensive health systems, are neither affordable nor practical for most populations globally. The guidelines also cover all aspects of care, from specific pharmacological, psychological, and social interventions, to general principles of care, including those related to autonomy and dignity. The mhGAP provides a robust foundation for scaling up by answering the key question *of what* should be scaled up. Now, the primary research challenge is addressing the *how* question—that is, how these treatments can be scaled up. The mhGAP-IG is currently being evaluated in Ethiopia, Jordan, Nigeria, and Panama as part of mhGAP implementation. Concurrently, the United Kingdom's Department for International Development has funded the Programme for Improving Mental Health Care (PRIME), a six-year research consortium led by the University of Cape Town, to adapt and evaluate the mhGAP-IG in primary health care settings in Ethiopia, India, Nepal, South Africa, and Uganda.

### Building Evidence on How Scaling Up Can Be Achieved Through Task Shifting

Only recently has it come to be accepted that the scope of “translational medicine” extends beyond its traditional focus on translating basic science discoveries into “products” with a potential to improve health, and that it requires, in addition, translating the knowledge of efficacious treatments into improved clinical practice and, ultimately, into improved health outcomes in entire populations. This process of translation will potentially encounter at least three roadblocks: between basic science and “first in man” studies; between efficacy and effectiveness trials; and between the formulation of clinical guidelines and the delivery in routine clinical practice.^23^ Implementation research is often considered to be concerned with the second and, in particular, the third of these roadblocks. The roadblocks lie on the path between knowing what works (as synthesized in the mhGAP-IG) and how it will be delivered “to scale”—that is, to entire populations. The single largest barrier to scaling up efficacious treatments for mental disorders is the enormous scarcity and inequality in the distribution of skilled human resources in low-resource settings.^16^ In simple numerical terms, one need only compare the number of psychiatrists in the United States (estimated as 50,000 for a population of roughly 300 million) with the number in India (estimated as 4000 for a population of well over a billion) to provide a vivid illustration of this iniquity. This vast disparity in relative numbers hides equally massive within-country disparities since the majority of psychiatrists in India work in urban areas, where only a minority of the population lives.

To close the treatment gap, mental health care must therefore be delegated to nonspecialist health workers who are trained to deliver interventions for specific mental disorders. Task shifting, which refers to the strategy of rational redistribution of tasks among health workforce teams, has become a popular method for addressing shortages of specialist health resources. When appropriate, highly qualified health workers share specific tasks with health workers having less training and fewer qualifications in order to make more efficient use of the available human resources. It is not surprising, then, that the leading research questions emerging from a priority-setting exercise (in the context of scaling up) were to develop and evaluate mental health interventions for delivery by nonspecialist health workers and to determine how such task-shifted interventions could be integrated within routine health care delivery systems.^24^ It is heartening to observe that several leading research funders have redoubled their commitment to supporting such implementation research to reduce the treatment gap. An excellent example of such a commitment is the recent National Institute of Mental Health (U.S.) initiative to support the establishment of “hubs” to promote research to reduce the treatment gap in LAMICs.

The evidence base for task shifting in mental health care in LAMICs, though comparatively young, is growing and is consistent in its findings. We now know that lay people or community health workers can be trained to deliver psychological and psychosocial interventions for people with depressive and anxiety disorders, schizophrenia, and dementia in a diverse range of LAMICs.^25^–^29^,^30^ A critical element of these task-shifting interventions—and a significant departure from earlier efforts to improve primary mental health care—is that the role of mental health specialists extends well beyond the training phase; they provide continuing supervision, quality assurance, and support to the nonspecialist health workers.^31^ This model is thus similar to “collaborative” models of care, which are the most effective delivery systems for depression management in primary care in developed countries.^32^ It may well be more appropriate to refer to such collaborative models of care as “task sharing” rather than “task shifting.” The most recent investigation of task sharing is the MANAS trial. That trial—the largest trial in psychiatry in LAMICs—demonstrated the effectiveness of trained lay counselors in improving recovery rates in patients with anxiety or depressive disorders at primary health care centers.^33^ By demonstrating the role of lay people in providing mental health care, trials like MANAS throw open the door of opportunity for task sharing to virtually anyone in the population.

### Building a Coalition for Mental Health: The Movement for Global Mental Health

The Movement for Global Mental Health (Movement), launched in October 2008, is a coalition of individuals and institutions committed to collective actions that aim both to close the treatment gap for people living with mental disorders worldwide and to promote their human rights—focusing on those populations with the largest gaps.^34^ A key goal is to provide the diverse range of parties involved—mental health professionals of all dispositions, civil society activists, global health advocates, and people affected by mental disorders—an opportunity to cast aside their differences, to stand shoulder to shoulder, and to advocate for a shared cause. Since its inception, the Movement has grown to over 1700 individual and 94 institutional members (as of 10 July 2011) from over 100 countries around the world, with strong participation of women, people from LAMICs, and people affected by mental disorders. The Movement seeks to build a coalition whose diverse members not only share a common goal but can use the Movement both to support their own activities and to strengthen the Movement itself. The website (http://www.globalmentalhealth.org) plays a central role as a “virtual headquarters.” Any individual and any institution that share these goals can become a member through the site, which also includes a wide range of resources submitted by members for sharing, such as packages of care, human rights stories, and advocacy articles. In early 2009, a profile of the Movement was added to the social-networking site Facebook, expanding the Movement's online presence. In 2010, the Movement decided that it needed a voluntary secretariat to facilitate its growth; through a participatory process, the Centre for International Mental Health of the University of Melbourne was selected as the secretariat for the first three-year term (2011–14). Major examples of the Movement's activities include hosting biennial summits as a platform for members to meet face-to-face and to share their experiences and initiate new collaborations (the second summit took place in Cape Town in October 2011); developing a Capacity Building Atlas for Global Mental Health (http://www.globalmentalhealth.org/cb_atlas.html), which provides information on relevant training programs; partnering with the *Lancet*, an institutional member of the Movement, to prepare and launch the series of six articles on global mental health published in October 2011;^35^ and partnering with the World Federation for Mental Health, another institutional partner, in support of its Great Push for Mental Health global campaign.^36^

### Addressing the “Grand Challenges in Global Mental Health”

A “grand challenge” in the context of global health is a specific barrier that, if addressed, would help to improve the lives of those affected by a health problem. The goal of identifying these challenges is to develop interventions that, if successfully implemented, would have a high likelihood of feasibility for scaling up and having a significant impact. The Grand Challenges in Global Mental Health Initiative (http://www.grandchallengesgmh.nimh.nih.gov), which follows two high-profile Grand Challenge initiatives (the first focused on infectious diseases, and the second on cardiovascular and metabolic disease), is led by a consortium comprising the Global Alliance for Chronic Diseases, National Institute of Mental Health, and Wellcome Trust. The initiative used a Delphi methodology, with over 400 global experts, to prioritize 25 “challenges.”^37^

Unsurprisingly, the leading five challenges—ranked according to their potential for reduction of disease burden, impact on equity, immediacy of impact, and feasibility— focused on improving access to evidence-based care and building the mental health skills of all health care personnel. Other challenges focused on improving our understanding of the root causes of, and protective factors for, mental disorders and on advancing knowledge that can lead to more effective prevention and early interventions.

Four broad themes were evident: (1) research needs to incorporate a life-course approach acknowledging the developmental origins of many mental disorders; (2) suffering related to mental disorders extends to families and communities, with the consequence that health system–wide changes are essential, along with efforts to reduce social exclusion and discrimination; (3) all care and treatment interventions must be grounded in evidence; and (4) mental health and environmental exposures, such as extreme poverty and war, are closely related.

As is evident from this initiative, both “discovery” and “delivery” research is needed to significantly reduce the burden of mental disorders worldwide.^37^ In the few months since the publication of the findings, two funders (Grand Challenges Canada and the Task Force for Neurosciences, Department of Biotechnology, [government of] India) have already announced calls for applications for research targeting these challenges.

## THE FUTURE

Global mental health is the discipline that seeks to address one of the most neglected global health issues of our time. It is also one of the most exciting and dynamic disciplines of global health, with a growing legion of advocates, donors, commitments, and initiatives. While we must celebrate this “coming of age,” the field still has a long road ahead—one strewn with challenges. Three major barriers loom ahead and will need to be addressed head-on.

The first barrier is the pervasive stigma against those who are living with mental disorders—which affect virtually every domain of their lives. Included here is the low political will of countries to prioritize and act on the evidence base of global mental health. Although some countries, such as Brazil and India, have shown some capacity to set their own health agendas independent of foreign donors and are now putting greater resources behind mental health,^18^ these allocations remain insufficient when compared to the scale of unmet needs. In countries whose health policies remain in the grips of international donors, the ability to prioritize mental health is hindered by the blinkered views of Northern donors, who generally harbor misguided views that mental health is not a priority for poor people or less-resourced countries. Activists from the Movement for Global Mental Health recently called for a special session of the UN General Assembly or for a summit on mental health^38^—which may prove to be a fruitful way of galvanizing the global community, from donors to governments, to act on the evidence that we already possess.

The second major barrier is the mental health community's relatively weak engagement with the agenda of global mental health. Psychiatry and other mental health professions have a key role to play in scaling up for several reasons: specialists need to provide the supervision needed for the successful implementation of task sharing; psychiatrists are often key decision makers for mental health policies and programs; and the leadership of psychiatrists is needed to encourage the broader profession to work in solidarity with other mental health stakeholders. A recent survey of leaders in psychiatry in nearly 60 countries showed their support for three strategies for reducing the treatment gap: increasing the numbers of psychiatrists and other mental health professionals; increasing the involvement of a range of appropriately trained nonspecialist providers; and increasing the active involvement of people affected by mental disorders.^39^ In the new world of global mental health, where an increasing proportion of front-line mental health care is shared with nonspecialist health workers, unique demands will be placed on psychiatrists and other mental health practitioners. They will need to be proficient in skills for training and supervising nonspecialist health workers; be engaged in monitoring and evaluation for quality assurance of mental health care programs; acquire the management skills essential for leading teams of health workers; and serve as advocates for the human rights of people with mental disorders. Put simply, the models used in comparatively better-resourced settings—with their armies of mental health professionals (which, ironically, still never seem sufficient to meet local needs)—have no chance of addressing the huge treatment gaps in LAMICs.^40^

The final barrier relates to the imperfections in our current state of knowledge about the nature of mental disorders and the armamentarium of effective treatments. The bottom line is that, when compared to what we know about most other chronic and noncommunicable diseases, we are far from understanding either the etiology of the perplexing disorders that affect mental health and also far from possessing treatments that are equally effective. It is clear that we need more investment in research into the nature and treatment of mental disorders, as highlighted by the Grand Challenges, and that this research must be carried out in both high-income countries and in LAMICs. Population-based studies should help us to characterize the phenotypes of mental disorders, as well as the variations in the distribution of disorders between and within diverse populations. The World Mental Health Surveys and the 10/66 Dementia Research Group program are two examples of such global, cross-national initiatives to study the prevalence and impact of mental disorders, along with health systems' responses to them. We need similar initiatives in areas not covered so far—notably, psychoses and child mental disorders. We also need to increase the pool of efficacious, safe, and acceptable treatments for mental disorders. Integrating strategies used in traditional systems of health care in non-Western cultures may be one option, as has been illustrated by mindfulness-based techniques (now adapted for use in cognitive and behavioral therapies). No systematic research initiatives have been undertaken, however, to map and evaluate the treatments, pharmacological or psychosocial, used by African, Asian, or native American cultures from time immemorial for managing mental disorders. Is the mental health equivalent of arteminisin awaiting discovery?

Where the field of global mental health will be ten years from now is difficult to predict, but the portents are promising. The developments in recent years have renewed the passion and commitment of advocates—academics, practitioners, and those who live with mental disorders and their families—to promote the cause of global mental health and to ensure that it remains in the foreground of global health efforts.
